# Exercise Mediates Heart Protection via Non-coding RNAs

**DOI:** 10.3389/fcell.2020.00182

**Published:** 2020-03-20

**Authors:** Yuelin Zhang, Nana He, Beili Feng, Honghua Ye

**Affiliations:** ^1^Department of Cardiology, HwaMei Hospital, University of Chinese Academy of Sciences, Ningbo, China; ^2^Ningbo Institute of Life and Health Industry, University of Chinese Academy of Sciences, Ningbo, China; ^3^Department of Experimental Medical Science, HwaMei Hospital, University of Chinese Academy of Sciences, Ningbo, China

**Keywords:** non-coding RNA, microRNA, exercise, cardiovascular diseases, oxidative stress

## Abstract

Cardiovascular diseases (CVDs) have become the central matter of death worldwide and have emerged as a notable concern in the healthcare field. There is accumulating evidence that regular exercise training can be as a reliable and widely favorable approach to prevent the heart from cardiovascular events. Non-coding RNAs (ncRNAs) could act as innovative biomarkers and auspicious therapeutic targets to reduce the incidence of CVDs. In this review, we summarized the regulatory effects of ncRNAs in the cardiac-protection provided by exercise to assess potential therapies for CVDs and disease prevention.

## Introduction

Cardiovascular diseases (CVDs) remain the most common cause of morbidity and mortality globally (Li et al., 2019). Hypertension, diabetes, high cholesterol, obesity, and alcohol and tobacco use are all risk factors for cardiac diseases. Despite advancements in therapeutic methods, the burden of CVDs has significantly increased given the high financial burden and associated health care problems they impose; thus, the development of innovative approaches to fight this major health problem is critical (Liu et al., 2017).

Regular exercise training (ET) has been considered to be the most effective intervention in preventing and reducing CVDs (Romeo et al., 2018). Exercise, which is considered the most effective, accessible, and inexpensive therapy a physician can prescribe, aids in blood pressure control, improves blood lipid profiles, and increases insulin sensitivity (Günay et al., 2018).

Numerous recent studies have demonstrated that ET-mediated heart protection involves compound interactions among multiple tissues and plays an intense role in gene expression (Malcolm et al., 2018). Emerging evidence suggests that non-coding RNAs (ncRNAs) set great effect on regulating cardiac-protection. In this review, we summarize the correlations among ET, ncRNAs, and CVDs to obtain a further insight of the molecular mechanisms underlying the physiological and pathological cardiac events with the hope of consequently offering brand-new targets for CVDs’ treatment.

## The Classification of ncRNAs

NcRNAs originate in the genome and are functional molecules that can not be translated to proteins. Based on molecular size, shape and function, they consist of certain different types, such as transfer RNAs (tRNAs), ribosomal RNAs (rRNAs), and short interfering RNAs (siRNAs), circular RNAs (circRNAs), piwi-interacting RNAs (piRNAs), and in much attention microRNAs (miRNAs, miRs) (Gomes et al., 2018; Dong et al., 2019). Notably, these numerous ncRNAs can regulate gene expression from several aspects such as epigenetics, transcription and post-transcriptional regulation, and thus participate in pathophysiological conditions as well as become central players in the occurrence and development of CVDs (Gomes et al., 2018).

MiRNAs are a class of non-coding RNAs with the length of 17∼22 nucleotides and can be in combination to the target gene’s 3′-untranslated region (UTR), thus in degradation of messenger RNA (mRNA) (Margolis and Rivas, 2018) and regulation of gene expression after transcription (Sapp et al., 2017). Each miRNA can bind to various target genes, while one same gene can also be conducted with a few of miRNAs (Zhang and Chen, 2018). It has been reported that miRNAs widely exists in humans, most of which have associations with tumor, inflammation and various diseases of human (Angel et al., 2019). Furthermore, miRNAs have been demonstrated that they can help further our understanding of responses to physical activity (Denham et al., 2018).

LncRNAs, a type of RNAs longer than 200 nt, have recently become a research focus given their functional importance and their ability to attenuate the inhibitory effect of miRNA on target genes and regulate expressions of target genes (Gomes et al., 2018).

CircRNA is defined as a closed cyclic form. Researches are being gradually revealed to analyze circRNA’s roles in the adjustment of gene expression (Bei et al., 2018; Zhang et al., 2018). However, the localization, degradation and biological functions of circRNA remain unclear (Bei et al., 2018).

## Exercise, Oxidative Stress, and ncRNAs

Oxidative stress refers to redox balance disturbances when the production of reactive oxygen species (ROS) exceeds the ability of self-clearance, conveying oxidative damage to cells and being the basis for the pathogenesis of numerous diseases. Elevated levels of ROS are not conducive to the utilization of cardiac calcium and related to endothelial dysfunction, acting as signaling molecules in the regulation of angiogenesis, necrosis and apoptosis (Tofas et al., 2019). Exercise as an important mediator of oxidative stress exerts two-sided effect on the regulation of the redox system. ROS generated by short-term physical activities is closely related to the formation of exercise adaptation. On the other hand, prolonged and intense exercise-induced oxidative stress could hinder skeletal muscle contraction and do harm to exercise capacity and body health (Di Meo et al., 2019).

Although there have been a lot of related researches on the relationship of free radicals and exercise adaptation and exercise-induced oxidative injury, the potential mechanisms are complex and insufficient studied. Kelch-like ECH associated protein 1 (*Keap1*)/nuclear transcription factor 2 (Nrf2) is a key signal pathway to resist oxidative damage. Exercise enhanced the expression of antioxidant enzymes and promotes cardiac antioxidant defenses by adjusting the Nrf2/*Keap1* ratio (Alves et al., 2020). Furthermore, during ET removal Nrf2 may activate stress-related kinases in white adipose tissue to impair insulin sensitivity, thereby altering glucose homeostasis (Merry et al., 2020). Moreover, ET could be able to promote sirtuin-1 *(SIRT1)* and better antioxidant activity in heart failure (HF) patients (Corbi et al., 2019). *SIRT1*, involved in the cellular response to exogenous stressors, is considered as sensor of oxidative stress and regulator of cell redox state (Conti et al., 2017).

Oxidative stress can also alter the expression of many ncRNAs. MiRNAs are in the most depth research since they set a huge impact on CVDs by inhibiting protein translation and targeting mRNA degradation. There is accumulating evidence that intracellular ROS can either suppress or induce miRNA expression levels via Nrf2, *SIRT1* and nuclear factor-kappa B (NF-κB) pathways (reviewed in Kura et al., 2020). However, further studies are still required to investigate and understand the underlying mechanism of ROS and ncRNAs during ET.

## Cardiac Changes in Response to Exercise

### Cardiac Hypertrophy and Cardiomyocyte Renewal

Cardiac hypertrophy (CH) is an adaptive compensatory condition to endurance exercise. The significance of exercise on the structure and capacity of the heart has being in topic interest currently (Xu et al., 2017). Thickening of the muscle forces the heart to work harder to circulate blood throughout the body (Samanta et al., 2016). CH can be classified into two forms: physiological hypertrophy and pathological hypertrophy. In contrast to exercise-induced physiological hypertrophy, characterized by changes in cardiomyocytes, thickening myofibrils, an increased number of mitochondria, and expansion of the sarcoplasmic reticulum to increase the cardiac reserve (Bei et al., 2017), pathological hypertrophy is a disease-related condition accompanied by apoptosis, necrosis and fibrosis that contributes to myocardial diastolic insufficiency, which reduces contractility (Gomes et al., 2018).

The pivotal role of miRNAs in cardiac growth has been in exploration in greater depth in recent years. MiR-17-3p was increased in hearts from mice that underwent 21 days of swimming training and was involved in exercise-induced hypertrophy, conceivably contributing to the protective impact of exercise (Shi et al., 2017). A previous study found that miR-17-3p directly and negatively targeted metallopeptidase inhibitor 3 (*TIMP3*) to strengthen cardiomyocyte proliferation and indirectly regulated the phosphatase and tensin homolog (*PTEN*), a natural inhibitor of the phosphoinositide 3-kinase (PI3K) (p110α)-protein kinase B (PKB/Akt) pathway [80], to stimulate physiological hypertrophy (Shi et al., 2017). Additionally, a negative relationship between changes in plasma miR-532 levels and insulin-like growth factor 1 (IGF-1) was observed in a previous study, which activating Akt signaling pathway to promote cell growth and proliferation as well as redox balance, thus promoting the adaptation to resistance exercise (Cui et al., 2017). After eight consecutive weeks of repeated running bouts, miR-1 expression decreased in exercised hearts, while mitochondrial calcium uniporter (*MCU*, a predicted target of the miR-1) protein levels increased (Zaglia et al., 2017). Therefore, the miR-1/*MCU* axis, controlled by the β-adrenergic receptor system, could affect mitochondrial Ca^2+^ uptake and was a factor in the dynamic adaptation of cardiac cells to hypertrophy (Zaglia et al., 2017). Another study demonstrated that inhibition of miR-222 restrained the homeodomain-interacting protein kinase 1 (*HIPK1*) (Vujic et al., 2018), a direct target of miR-222 with antiproliferative effects in cardiomyocytes (Ding et al., 2017), leading to the regulation of cardiomyocyte generation after exercise (Vujic et al., 2018).

MiR-1 was increased, and NCX1, the essential transport factor of Ca^2+^, was decreased in pathological hypertrophy in control obese rats (OZRs) (Silveira et al., 2017). Moreover, a decrease in miR-29c in OZRs exerted a positive effect on the cardiac collagen volumetric fraction (CVF) (Silveira et al., 2017). However, cardiac miR-29c and miR-1 levels were standardized through aerobic exercise training and eventually decreased collagen expression (Soci et al., 2011), pathological cardiac alteration and dysfunction, suggesting that AET exert a positive effect against pathological CH.

### Angiogenesis

A considerable relationship between endurance exercise and increased neovascularization or angiogenesis has been studied in depth (Liu et al., 2017). MiR-126, known as an endothelial-specific miRNA, targeting sprouty-related protein 1 (*SPRED1*) and phosphatidyl-inositol 3-kinase regulatory subunit 2 (*PIK3R2*), activated ERK and Akt, pathways to enhance the role of vascular endothelial growth factor (VEGF) and thus promote the process of angiogenesis (Fernandes et al., 2017). MiR-210, a hypoxia-specific miRNA, may stimulate neovascularization by downregulation of its target gene, *ephrin A3*, which is an important molecule in VEGF-mediated angiogenesis signaling pathway (Ghorbanzadeh et al., 2017; Naderi et al., 2019). After 8 weeks’ voluntary exercise, miR-126, miR-210, Akt, and ERK1/2 in cardiac tissue were upregulated in both the crocin and voluntary exercise groups (Ghorbanzadeh et al., 2017). Another study found that in the diabetic animals there was an decrease of miR-126 and angiogenesis, while the expression level of miR-210 was increased (Naderi et al., 2019). miR-16 decreased, and miR-21 increased after a 5 weeks’ high-intensity interval training (HIIT) intervention scenario (Horak et al., 2018). In addition, targeting VEGFR2 and fibroblast growth factor receptor 1 (FGFR1), miR-16 regulated the reduction in proliferation, migration and angiogenic behavior in endothelial cells (ECs) by while its overexpression led to reduced proliferation, migration and cord formation of ECs *in vitro* (Chamorro-Jorganes et al., 2011). miR-21 could be indirectly engaged in angiogenesis by promoting hypoxia inducible factor-1 (HIF-1α) and VEGF expression and thus may be an exceptional biomarker for evaluating the response to physical exercise (Sheedy, 2015).

### Anti-inflammation

Overexpression of miR-181b-5p in vascular endothelium has been shown to inhibit NF-κB signaling pathways by directly targeting importin-α3 expression to reduce inflammation/injury, thus serving as a regulator of inflammatory and immune responses (Sun et al., 2012; Cui et al., 2017). As mentioned above, the expression of miR-21 was increased during HIIT (Horak et al., 2018), mediating the anti-inflammatory response in macrophages and making it a novel and elegant target for therapeutic intervention (Sheedy, 2015).

### Antifibrosis

Regular exercise training can effectively inhibit oxidative stress, inflammation and apoptosis, thereby preventing the loss of cardiomyocyte and the formation of ventricular fibrosis (Darband et al., 2019). MiR-29a and miR-101a have been reported to increase with the expression of fibrotic proteins and downregulate with controlled intermittent aerobic exercise in infracted hearts, suggesting that miR-29 and miR-101 can be recognized as fibrosis-associated miRNAs (Xiao et al., 2017). By targeting transforming growth factor β 1 (TGFβ-1) or fos, upregulated miR-29a and miR-101a can prohibit myocardial fibrosis modulated by *COL1A1* and *COL3A1*, ultimately protecting myocardial cells from fibrosis and scar tissue formation (Xiao et al., 2017).

### Atrial Remodeling

The expression of miR-1 and miR-133a is increased immediately after marathons, and a negative correlation has been observed in the expression levels of miR-1 and miR-133a with left atrial (LA) diameter immediately after and 24 h after marathons in elite runners (ERs) (Clauss et al., 2016). That research implied that endurance exercise would influence energy metabolism (Clauss et al., 2016) and the increase of miRNAs reflect a latent mechanism behind the observation that mitochondrial dysfunction could affect miRNA expression (Baumgart et al., 2015). Circulating miRNAs could be characterized as potential and novel biomarkers for atrial remodeling.

## MiRNAs Respond to Cardiac-Protection by Exercise

The correlation between exercise and CVDs has been in popularity for decades. Several typical signal pathways and molecular mechanisms of miRNAs have been proposed (Table 1), which offer an innovative prospect for cure.

**TABLE 1:** Changes of miRNAs after exercise in CVDs.

Diseases	Exercise types	MiRNAs	Expressions	Targets	Sources
Coronary artery disease	Swimming	miR-20a (Wang et al., 2017)	Increased	*PTEN*	Endothelial cells
Myocardial infarction	Intermittent aerobic exercise	miR-29a and miR-101a (Xiao et al., 2017)	Increased	TGFβ-1 and fos	Heart tissue
Ischemia-reperfusion	Swimming	miR-21 (Zhao and Ma, 2016)	Increased	*PDCD4*	Cardiomyocytes
	Swimming and running	miR-17-3p (Shi et al., 2017)	Increased	*TIMP3*	
Heart failure	Cycling	miR-21,	Increased	*PPAR*?	Serum
		miR-378, and		ATP6?	
		miR-940 (Xu et al., 2016)		Not mentioned	
	Wheel running	miR-222 (Vujic et al., 2018)		*HIPK1*	Cardiomyocyte
Hypertension	Exercise training	miR-34a and miR-181a (Fernandes et al., 2017)	Decreased	*SIRT1*	Heart tissue
	Running	miR-145 (Liao et al., 2018)	Increased	*IRS-1*	Arteries
		miR-214 (Rodrigues et al., 2018)		SERCA2a	Cardiomyocytes
Pulmonary hypertension	Exercise training	miR-22-3p relative to miR-451a (Grunig et al., 2018)	Decreased	Not mentioned	Serum and plasma
Cardiometabolic diseases	Strength training	miR-146a (Morais Junior et al., 2017)	Increased	*TRAF6*	Serum

### Exercise Mediates Protection Against Atherosclerosis and Myocardial Infarction

Atherogenesis (ATH) is a chronic disease of blood vessels and involves various factors, such as inflammation, immune mechanisms and lipid infiltration, leading to many complications, such as myocardial infarction (MI) and stroke (Libby et al., 2011). MI occurs when the coronary artery undergoes acute persistent ischemia and hypoxia, resulting in myocardial necrosis and pathological cardiac remodeling.

Recently, the miRNA expression profile revealed that a series of miRNAs shows great importance in ATH and MI. Epicardial adipose tissue thickness is reported to be one of the major risk factors of coronary artery disease (CAD), and miR-20 is correlated with adipogenesis (Isabelle et al., 2015). A recent study occurred that miR-20a was enhanced by swimming training in CAD mice. Therefore, A group of associated atherosclerosis genes was regulated, endothelin 1 (*EDN1*), angiotensin II (ANGII), as well as thromboxane A2 (TxA2) downregulated and endothelial nitric oxide synthase (eNOS), prostacyclin (PGI2), and VEGF upregulated (Wang et al., 2017). Increased miR-20a suppressed the expression of *PTEN*, which eventually contributes to increased survival and proliferation of venous ECs through stimulating of PI3K/Akt signaling pathways (Wang et al., 2017). Furthermore, as previously mentioned, after MI, upregulated miR-29a and miR-101a restrained the TGFβ-1/Smad2/3 and fos/TGFβ-1 pathways via aerobic exercise, eventually inhibiting myocardial interstitial fibrosis (Xiao et al., 2017). This finding could lead to a potential therapeutic strategy for cardiac-protection in MI patients. In addition, miR-92a played a pivotal role in angiogenesis and was associated with arterial dysfunction. miR-92a was reported to be downregulated in aging and its outcomes were relevant to increases in major arterial structural proteins, such as type 1 collagen, and proinflammatory receptors, such as tumor necrosis factor receptor 1 (*TNFR1*) (Hazra et al., 2016). Another study demonstrated that miR-92a increased in the process of endothelial injury after acute myocardial infarction (AMI) and inhibited Kruppel-like factor 2 (*KLF2*) and *KLF4* expression (Liu et al., 2016). However, little information is available about the association between exercise and miR-92a. Thus, further studies should be under consideration to assess the miR-92a’s functional impacts of exercise.

### Exercise Mediates Protection Against Ischemia-Reperfusion Injury (IRI)

Tissue damage induced by ischemia is the leading cause of fatal diseases. In the therapeutic treatment of ischemic diseases, tissue damage is primarily induced by restoration of the recovered blood supply, which contains excessive free radicals, rather than the ischemia itself. Increased ROS can modify cellular signaling proteins and produce functional consequences, thereby mediating the pathological processes involved in the development of IRI.

The heart may undergo an increase in apoptosis during ischemia-reperfusion (I/R) (Duan et al., 2012). Swimming training increased miR-21 levels and decreased programmed cell death protein 4 (*PDCD4*) expression (Zhao and Ma, 2016). miR-21 downregulated *PDCD4* expression, a critical mediator of cancer cell apoptosis, and cardiomyocyte apoptosis induced by I/R was consequently aggravated (Cheng et al., 2010). Although a remarkable correlation between swimming training and the expression of *PDCD4* didn’t be observed, that study still indicated that swimming training was beneficial to exercise-mediated cardiac-protection (Zhao and Ma, 2016). As we described above, miR-17-3p was proven to participate in exercise-induced CH. The study also found that the expression of miR-17-3p was efficiently increased in the hearts of both sham and IRI mice, implying that increasing miR-17-3p levels effectively prevent from myocardial IRI (Shi et al., 2017). Compared with the control group, miR-22 is significantly increased in the heart of rats suffered from IRI in reaction to the following oxidative stress (Du et al., 2016). MiR-22 has been proven to potentially regulate *SIRT1* and peroxisome proliferator-activated receptor-coactivator-1α (PGC1α), both of which are proven to improve mitochondrial function and inhibit oxidative stress, thus alleviating MI (Du et al., 2016). However, the role of miR-22 in exercise needs to be further explored.

### Exercise Mediates Protection Against HF

HF is a term used to describe a heart that cannot keep up with its workload, resulting in the body not receiving the oxygen it needs. The expression of miRNA species in HF and the accompanying proteomic remodeling has been characterized previously (Pinti et al., 2017). Oxidative stress plays a critical part in the development of HF. Elevated ROS causes systolic failure and structural damage, leading to myocardial dysfunction (Shirakawa et al., 2019). Exploration of the molecular mechanisms of miRNAs is still in progress.

MiR-21 targeted and suppressed the peroxisome proliferator-activated receptor (*PPAR*) transcript, which regulates plays an important regulatory role in inflammation, ATH, tumors, and fatty acid anabolic, was shown to be increased during HF (Lai et al., 2015; Pinti et al., 2017). Overexpression of miR-378 suppressed ATP synthase F0 subunit 6 (ATP6) and was characterized in the interfibrillar mitochondria amidst diabetes mellitus (Jagannathan et al., 2015; Shepherd et al., 2017; Li et al., 2018). Recent studies showed that cardiopulmonary exercise test was fairly able to elevate the serum levels of miR-21, miR-378, and miR-940 in HF patients (Xu et al., 2016). Although failed to reveal whether the modulation of these miRNAs was associated with acute exhaustive exercise, muscle damage or inflammation, the authors speculated that exercise may release these miRNAs into the circulation by inducing changes in cells nearby or in distant tissues (Xu et al., 2016).

### Exercise Mediates Protection Against Hypertension

Hypertension, one of the most common chronic diseases, is the most crucial risk factor for cardiovascular and cerebrovascular diseases. Getting effective command of blood pressure could significantly reduce cardiovascular and cerebrovascular events and enhance quality of patients’ lifespan (Improta Caria et al., 2018).

Compared with the sedentary group, the group of rats that underwent ET exhibited a decrease in aortic miR-34a and miR-181a expression, and hypertension was associated with increases in these miRNAs in spontaneously hypertensive rats (SHR) (Fernandes et al., 2017). miR-34a prohibiting *SIRT1* was found to be in the acceleration of endothelial progenitor cell senescence (Zhao et al., 2010). Moreover, low content of NO is associated with endothelial dysfunction and contributes to the maintenance and development of hypertension. Isometric exercise was reported to increase pro-oxidant activity, which resulted in greater NO bioavailability and antioxidant response, thus reducing patients’ blood pressure (Olher et al., 2019). The regulation of eNOS/NO system, *SIRT1* activated endothelium-dependent vascular relaxation, thus exhibiting significant protective effects in cellular aging (Ilwola et al., 2007). A previous study showed that miR-34a and miR-181a targeted *SIRT1* in the heart but did not include any discussion about eNOS (Fernandes et al., 2017). The abnormal proliferation of vascular smooth muscle cells (VSMCs, which exert important functions in structural remodeling) are the main pathological basis for hypertensive vascular remodeling and the development of vascular proliferative diseases. MiR-145 abounds in normal arteries, especially in VSMCs. Compared with control groups, miR-145 in spontaneously hypertensive rats was lower and exercise promoted both the levels of miR-145 and Akt phosphorylation (Liao et al., 2018). MiR-145 was also found to target insulin receptor substrate 1 (*IRS1*) and thus regulate the Akt signaling pathway (Liao et al., 2018). Interestingly, another study showed that in hypertensive rats, ET reduced systolic arterial pressure and increased the accessibility of intracellular Ca^2+^ in myocytes of the left ventricle as well as the expression of miR-214 (Rodrigues et al., 2018). However, the results contradicted the author’s expectations and data from previous research that revealed decreased miR-214 expression and increased expression of sarcoplasmic reticulum Ca^2+^-ATPase (SERCA2a), the main facilitator of Ca^2+^ translocation from the cytosol to the sarcoplasmic reticulum (Melo et al., 2015). Thus, further studies are needed to investigate the possible effects of aerobic exercise on hypertensive cardiomyocytes.

### Exercise Mediates Protection Against Pulmonary Hypertension

The main feature of pulmonary hypertension (PH) is a progressive increase in pulmonary artery resistance and the right heart, causing pulmonary vascular remodeling and constriction. ET has been a brand new type of therapy to help PH patients improve their mobility and quality of life. ET quite declined expression levels of miR-22-3p relative to those of miR-451a in serum (Grunig et al., 2018). That research highlights that miRNAs were to be future biomarkers, though it did not disclose the molecular mechanisms of miRNAs in response to ET in the pulmonary vasculature (Grunig et al., 2018).

### Exercise Mediates Protection Against Cardiometabolic Diseases

The aging process leads to many physiological and pathological changes in the human body, such as dysfunction of organs, loss of muscle mass and imbalance of metabolism (Margolis et al., 2016), thus in danger of cardiometabolic diseases (CMDs). Type 2 diabetes mellitus (T2D) is an eminently predominant metabolic disease that can result in progressive cardiac dysfunction (Hathaway et al., 2018), contributing to an independent risk factor of CVDs (Improta Caria et al., 2018). Aerobic exercise was reported to effectively protect the heart from accumulation of ROS and may have a therapeutic effect on diabetic cardiomyopathy (Wang et al., 2020). MiR-146a was increased after strength training, reducing serum blood glucose levels in patients with diabetes compared with those without (Morais Junior et al., 2017). miR-146a represses TNF receptor associated factor 6 (*TRAF6*), an element of innate and adaptive immunity that serves as an activator in the NF-κB pathway (Morais Junior et al., 2017), to not only dominate the expression levels of inflammatory gene but also limit the shift from “oxidative phosphorylation to glycolytic metabolism during inflammation” (Runtsch et al., 2019).

## LncRNAs and circrnas Respond to Cardiac-Protection by Exercise

LncRNA microarray analysis has been in depth use to measure the consequence of exercise in the expression of lncRNA and mRNA. A recent study showed that swimming training might alleviate vascular IRI via FR030200-Col3A1 and FR402720-Rnd1 compared with consumption of a high-fat diet by improving insulin sensitivity (Liu et al., 2018). FR030200-Col3A1, FR402720-Rnd1, and FR030200/FR402720-Rnd3 signaling were identified in the cytoskeletal rearrangement pathway, while E2F1-FR030200/FR402720-Nnat and FR030200/FR402720-Fam46a signaling in the anti-inflammatory process (Liu et al., 2018). A previous study identified the latent correlation between lncRNA-mRNAs and ET as novel biomarker as well as therapeutic strategy for vascular endothelial IRI. Furthermore, lncRNA THRIL, as a transcriptional regulator of TNF-α, was altered after a half marathon race and was speculated beyond of NF-kB signaling pathway (Gary et al., 2019).

Differing from miRNAs and lncRNAs, circRNAs have been studied for only the past several years. A vast majority of complex functions and mechanisms of circRNAs remain unclear, with most research focusing on determining their roles in various diseases (Bei et al., 2018; Zhang et al., 2018). Studies of the connections of circRNAs between exercise and cardiac-protection are still in their infancy.

## Summary and Future Perspectives

**FIGURE 1:**
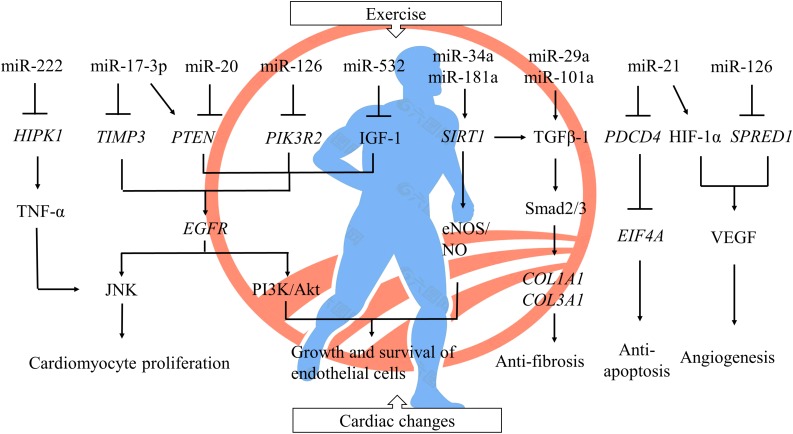
The regulation of miRNAs to cardiac changes after exercise. A single miRNA could have thousands of target genes, and one and the same gene could also be conducted by a host of miRNAs. Exercise-induced cardiac protection involves a series of complex regulation between miRNAs and their signal pathways by inhibiting protein translation and targeting mRNA degradation. ⊥ inhibited; Gene symbols were italicized. *HIPK1*, homeodomain-interacting protein kinase 1; TNF-α, tumor necrosis factor α; *TIMP3*, metallopeptidase inhibitor 3; *PTEN*, phosphatase and tensin homolog; *PIK3R2*, phosphoinositide-3-kinase regulatory subunit 2; IGF-1, insulin-like growth factor 1; *EGFR*, epidermal growth factor receptor; JNK, c-Jun N-terminal kinase; PI3K, phosphoinositide 3-kinase; *SIRT1*, sirtuin-1; NO, nitric oxide; eNOS, endothelial nitric oxide synthase; TGFβ-1, transforming growth factor β 1; *COL1A1*, collagen type I alpha 1 chain; *COL3A1*, collagen type III alpha 1 chain; *PDCD4*, programmed cell death protein 4; *EIF4A*, eukaryotic translation initiation factor 4A; HIF-1α, hypoxia inducible factor-1; *SPRED1*, sprouty-related protein 1; VEGF, vascular endothelial growth factor.

In this review, we have probed into the molecular mechanisms of ncRNAs about exercise-mediated protection of CVDs. MiRNAs, stable in expression, transferred and communicated through extracellular vesicles and released into circulation to accomplish multiple biological functions, have a positive effect on cardiac-protection via targeting related genes in numerous signaling pathways (Figure 1), thus becoming the promising biomarkers and targets for diagnosis and treatment of CVDs. LncRNAs regulate expressions of target genes and their signaling pathways in multiple links. However, the roles of circRNAs require further research.

Exercise-derived oxidative stress is one of the most important factors in development of CVDs. There is an interaction between ncRNAs especially miRNAs and ROS, but we still have a finite awareness of the molecular mechanisms of miRNAs regulating CVDs under ROS-related stress conditions. Moreover, it remains challenging to translate the results of recent studies of CVDs to clinical applications. Since this field is only in its early stages, researches on exercise-mediated ncRNAs have been generally based on miRNAs. However, the expression of other ncRNA families in exercise are still in lack of relevant exploration. Above all, these preliminary studies provide an optimistic perspective of ncRNA-based therapeutics for cardiac rehabilitation.

## Author Contributions

HY designed the theme of the review. YZ wrote the manuscript. NH, BF, and HY reviewed the manuscript. HY approved the final edition.

## Conflict of Interest

The authors declare that the research was conducted in the absence of any commercial or financial relationships that could be construed as a potential conflict of interest.
